# Natural Gas

**Published:** 1887-10

**Authors:** Joseph D. Weeks


### NATURAL GAS.

On the western shore of the Caspian, a narrow tongue of land but twenty miles broad from sea to sea, thrusts itself far out into the waves of this remnant of the great ocean, that once covered the steppes of the Ural and Volga. It is the Aspheron Peninsula, the continuation of the mighty Caucasus Mountains, as they plunge beneath the sea. From this peninsula, as well as on the islands that stretch beyond it, and even from the sea itself, strange lights have flared for centuries. When they were first lighted, no man can tell ; but as they flamed on through ages, fed by a mysterious and inexhaustible fuel, is it a wonder that tradition held that they were lighted by Noah, as he came down from the neighboring Ararat, and that prophecy foretold that they would burn on to the end of the world ?

For 2,500 years at least, this flame has been burning, and during all this time, so it is asserted, it has lighted the prayers of the priests of the purest religion known to the heathen world—the Fire Worshippers. Here they built an altar, and upon it, through all these centuries, a long succession of priests has tended the sacred flame with holy ardor and watchful care. To them it was the fire symbol of the eternal and omnipotent God they worshiped, and to them and to the awe-struck votaries of their mysterious faith, the region became known as the "Land of Eternal Fire."

But the demands of modern industry have overthrown the altar, and driven its priests from its side. The vigil is at an end. The eternal fire has gone out, but in another and far-off land it has been rekindled, not as a symbol of worship, but to bring warmth and cheer to more than twice ten thousand Christian homes; for the mystic flame of the Caspian, before which the Magi bowed in silent awe, was the fire-light from the same natural gas that burns to-day in so many of the homes of this Western world.

Natural gas, it thus appears, is no new product. Three thousand years ago, the Chinese found gas three thousand feet below the earth's surface, when drilling salt wells, and have been piping it through bamboo pipes, just as we do through iron ones, and burning it in clay burners as we burn it in lava tips or brass. Cæsar warmed his shivering hands at the glowing flame of the Fontaine Ardente, in Gaul, with the same satisfaction and comfort, that many a sovereign of this Western republic experienced in warming his, at his gas fire the cold mornings last winter.

Nor has the natural gas neglected to give mankind frequent intimations of its awful power in these years of the past. The deadly fire-damp, that tells in the dread rumble and the quivering earth that death and destruction are abroad in the mine, is the same natural gas that we take into our workshops and homes, and which, like a willing giant, serving, not ruling, does willing useful work.

When natural gas first made its presence known in this country cannot be stated. The Indians, and possibly the Mound Builders before them, must have had knowledge of some of the many surface indications, of the leaks from the gas reservoirs that are so common in the valleys of the upper Ohio, and which for centuries have told of the existence of this gas. Unlike its twin brother, petroleum, it was not born to this upper world but to pass away at its birth. It took no recognizable part in those striking and mysterious scenes, the petroleum burning at weird midnight over the waters of Oil Creek, which so impressed the early French missionaries as they journeyed down the Alleghany to the Ohio.

However, there are records of its presence here, going back more than a hundred years. The burning spring in the Kanawha Valley of West Virginia, which once belonged to Washington, is one of the earliest known recorded sources of gas in this country. In 1821, the little village of Fredonia, New York, was lighted with gas from a shallow well, and a little after, the light-house at Barcelona, a harbor on Lake Erie. Twenty years after Fredonia's first use, a salt manufacturer of the Kanawha Valley burned it under his "salt blocks." As early as 1838, it was used in a dwelling house in Findlay, O. From an early date in the history of the development of the oil region of Pennsylvania, the gas, which generally accompanies the oil, has been used in drilling wells, pumping oil, and for light and heat in the towns and villages near the wells. Indeed, until 1883, few wells had been bored for gas. Nearly all gas wells had been struck while boring for petroleum.

Notwithstanding these earlier uses, it was not until the introduction of the gas from the Murrysville well into Pittsburg, but three years ago, that natural gas began to assume the importance as a fuel which it now possesses. At that time its future was not dreamed of. Two or three rolling mills, glass works here and there, possibly a score of industrial establishments, all told, and a few dwelling houses used the gas for fuel. To-day it cooks the food of thirty thousand families and warms as many homes ; it puddles the iron and rolls the steel ; it melts the glass, it burns the pottery ; it drills the wells and pumps the oil and refines it ; it furnishes carbon for ink, for paint, and for electric lamps ; it raises the steam in many thousand industrial works. In a word, it is the fuel for domestic purposes and for use in the arts wherever it can be obtained, and so much superior is it to coal, that cities with coal at their very doors, pipe the gas sixty or seventy miles for use in their homes and workshops.

And what is this natural gas ? There is a remarkable series of compounds of hydrogen and carbon known as the paraffines. Some of these are solid at ordinary temperatures, as paraffine wax, others are liquid, while still others are gaseous. Our American petroleum is composed almost entirely of liquid paraffine, holding solid paraffine in solution, while natural gas, which is so intimately associated with petroleum as to be scarcely, if ever, absent when that is present, is chiefly the first of the series of gaseous paraffines, methane ($C\,H_4$), the marsh gas of the stagnant pool, the light, carbureted hydrogen of the chemist, the explosive fire-damp of the miner. With this marsh gas is mixed quite a number of other gases, chiefly ethane, another of the paraffines, considerable hydrogen, and, at times, nitrogen, a little olefiant gas, the illuminating gas of our cities, with small amounts of carbonic oxide, carbonic acid and oxygen.

Analyses given seem to show that the proportions of the several gases vary most remarkably. This is one of the most interesting and inexplicable facts in connection with natural gas. The gas from the same well, coming from the same storehouse, will, in two different ways, show most marked changes in composition. Four samples of gas were taken from the same well near Pittsburgh, on four different days. In one of these samples there was but forty-nine and one half per cent of marsh gas, in another seventy-two and one-fifth per cent., while the hydrogen in the two samples was thirty-six per cent. and twenty and five-eighths per cent, respectively. It is no wonder that with this great variation in composition the gas does not give as great a heat at some times as at others.

Usually the gas has little or no odor. This is one of the dangers connected with its use. It might escape into a room in sufficient qnantities to form an explosive mixture, without indicating its presence. There is, however, a slight odor to the gas when burning, that cannot be described, but which is soon recognized by those using it. Some gas has a distinct smell of petroleum, while that from certain deposits, the Findlay, for example, very soon announces its presence by a marked odor of ancient eggs, caused by the sulphureted hydrogen it contains.—*Joseph D. Weeks, in the Chautauquan.*

